# Inhaled corticosteroids do not affect the antibody titer against the SARS-CoV-2 spike protein in BNT162b2 mRNA vaccinated patients

**DOI:** 10.1186/s13223-022-00719-6

**Published:** 2022-08-25

**Authors:** Takeo Nakajima, Tatsuya Nagano, Yoshiharu Miyata, Shoko Murakami, Satoshi Mitsuyuki, Yohei Funakoshi, Kimikazu Yakushijin, Hitoshi Horimoto, Yoshihiro Nishimura, Kazuyuki Kobayashi

**Affiliations:** 1 Nakajima Medical Clinic, 1-8-3 Mikagenakamachi, Higashinada-ku, Kobe, 658-0054 Japan; 2 Division of Respiratory Medicine, Department of Internal Medicine, Kobe University Graduate School of Medicine, 7-5-1 Kusunoki-cho, Chuo-ku, Kobe, 650-0017 Japan; 3 Division of Bioresource Research and Development, Department of Social/Community Medicine and Health Science, Kobe University Graduate School of Medicine, 1-5-1 Minatojimanakamachi, Chuo-ku, Kobe, 650-0047 Japan; 4 Department of Hematology, Kobe City Medical Center General Hospital, 2-1-1, Minatojimaminamimachi, Chuo-ku, Kobe, 650-0047 Japan; 5 Division of Medical Oncology/Hematology, Department of Medicine, Kobe University Graduate School of Medicine, 7-5-1 Kusunoki-cho, Chuo-ku, Kobe, 650-0017 Japan; 6 Horimoto Clinic, 4-4-23, Okamoto, Higashinada-ku, Kobe, 658-0072 Japan; 7 Department of Respiratory Medicine, Kitaharima Medical Center, 926-250 Ichiba-cho, Ono, Hyogo 675-1392 Japan

**Keywords:** Inhaled corticosteroid, SARS-CoV-2, BNT162b2 mRNA vaccine, Asthma

## Abstract

**Objectives:**

Oral corticosteroids reduce the antibody titer of the BNT162b2 mRNA vaccine against SARS-CoV-2. To date, the effect of inhaled corticosteroids on antibody titers is unknown.

**Study design:**

The design of this study is retrospective study.

**Methods:**

We analyzed the relationship between the clinical features and total antibody titers against severe acute respiratory syndrome coronavirus 2 (SARS-CoV-2) spike protein in 320 subjects who had never been infected with Coronavirus disease 2019 (COVID-19) and were vaccinated the second time with the BNT162b2 mRNA vaccine between October 1 to December 28, 2021.

**Results:**

Of the 320 subjects, 205 were treated with inhaled corticosteroids. The median antibody titer of patients treated with inhaled corticosteroids was 572 U/mL, which was significantly higher than that of patients treated without inhaled corticosteroids (454U/mL, P = 0.00258). The median antibody titers of smokers, men, and patients aged 65 years and over, were 315.5 U/mL, 385 U/mL, and 425.5 U/mL, respectively. These results are significantly lower than those of patients who never smoked, women, and patients aged less than 64 years (582 U/mL [P < 0.0001], 682.5 U/mL [P < 0.0001], and 717 U/mL [P < 0.0001], respectively). The multivariate analysis revealed that females and age were independent antibody titer-reducing factors (P = 0.0001 and P < 0.0001, respectively).

**Conclusions:**

The use of inhaled corticosteroids did not reduce the antibody titer against SARS-CoV-2 spike protein. Clinicians should continue treatment with inhaled corticosteroids if indicated.

## Background

Coronavirus disease 2019 (COVID-19) is a pandemic affecting more than 250 million people worldwide. So far, more than 5 million people have died from the disease [1]. Various COVID-19 therapeutic agents have been developed. At the same time, vaccines for preventing COVID-19 have also been developed at a rapid pace [2]. Since clinical trials of vaccines evaluate immunogenicity in healthy individuals, immunogenicity in patients with concurrent diseases is not well understood. However, some diseases that weaken immunogenicity have been studied. The results of these studies suggest that antibody titers are low in patients on immunosuppressive medications (P < 0.001), the elderly (P < 0.001), alcohol drinkers (P = 0.037), and patients on glucocorticoids (P = 0.020) [3]. In a previous study, we reported that immunosuppressant agents, such as anti-CD20 antibody therapy for malignant lymphoma, lowered the antibody titer [4]. Although it is thought that biologics may be effective in asthmatic patients with COVID-19 [5], biologics themselves have been reported to lower antibody titers [6]. The Global Initiative for Asthma (GINA) 2021 guidelines recommend continued inhaled corticosteroids (ICSs) and COVID-19 vaccination for patients with asthma [7]. However, the effect of ICSs on vaccine antibody titers is unknown. Therefore, this study aimed to clarify the effect of ICSs on COVID-19 vaccination.

## Methods

### Patients

This study included 326 patients who received the second BNT162b2 mRNA vaccine between October 1 to December 28, 2021, at Nakajima Medical Clinic. Of the 326 patients, we excluded five patients who reported to be infected with COVID-19 and one patient who received anti-CD20 antibody therapy for malignant lymphoma. The ICS group had 2 patients with systemic corticosteroid and 1 patient with immunosuppressive medication. The non-ICS group did not have the patients with systemic corticosteroid and immunosuppressive medication. There were no patients with immunodeficiency in each group. In total, 320 patients were analyzed. This study was approved by the Review Board of the Hyogo Prefecture Medical Association (R3-008). Informed consent was obtained using the opt-out method, meaning the patients can decide not to participate in this study.

### Antibody titer quantification

The blood samples were collected 4 months after vaccination. For each patient, 2 ml of blood sample was sent to the LSI Medience Corporation for the quantification of the antibody titer against the severe acute respiratory syndrome coronavirus 2 (SARS-CoV-2) spike protein (total antibody) (Roche, Basel, Switzerland).

### Diagnostic definitions

Cough-variant asthma and cough-predominant asthma were diagnosed according to criteria described elsewhere [8]. Briefly, cough-variant asthma is characterized by (i) cough lasting more than 3 weeks without wheezing and (ii) cough that responds to bronchodilators. Cough-predominant asthma was diagnosed if the cough was accompanied by obvious wheezing on chest auscultation. Allergic rhinitis was diagnosed using the Self-Assessment of Allergic Rhinitis and Asthma (SACRA) Questionnaire [9].

### ICS dosages

The budesonide dosage was classified into low-dose (250–499 mcg/day), medium-dose (500–1199 mcg/day), and high-dose (> 1200 mcg/day). Similarly, the fluticasone dosage was classified into low-dose (100–299 mcg/day), medium-dose (300–499 mcg/day), and high-dose (> 500 mcg/day).

### Statistics

**Fig. 1:**
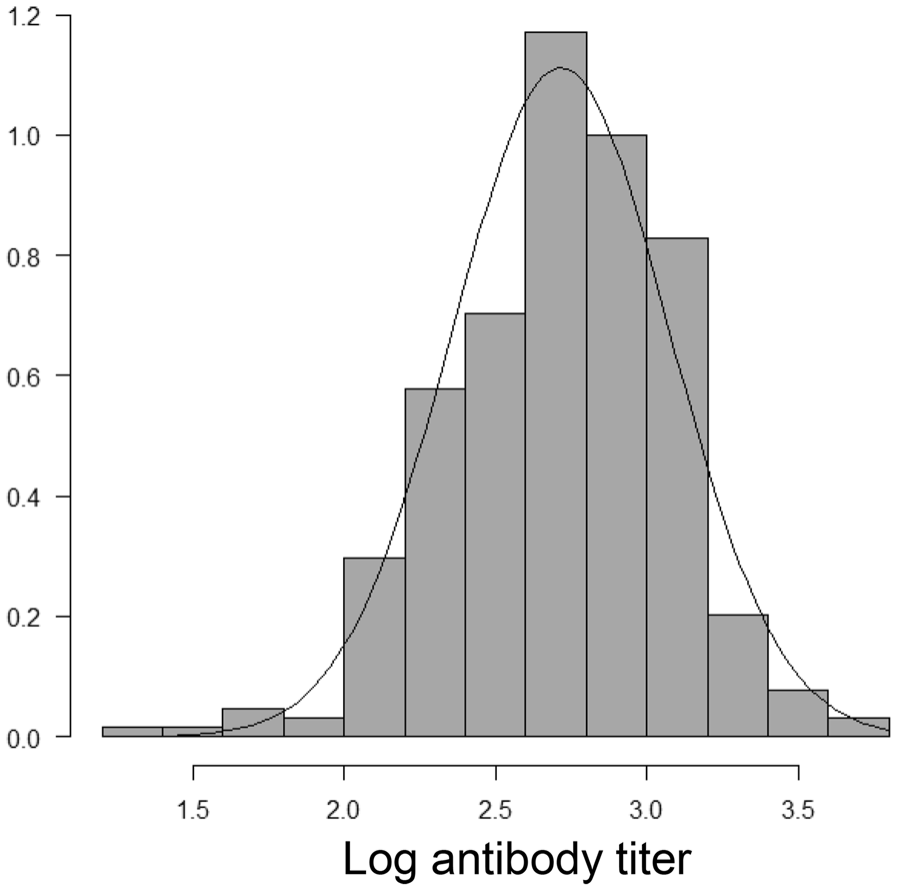
Log converted antibody titer follows the normal distribution

All statistical analyses were performed using EZR version 1.37 [10]. Differences in patient characteristics between the two groups were analyzed using Pearson’s χ^2^ tests or Fisher’s exact tests. For the univariate analysis, differences were assessed using the Mann–Whitney U test. Comparisons of three or more groups were performed using the Kruskal–Wallis test. Pairwise comparisons were performed using the Mann–Whitney U test. A multivariate regression analysis was conducted using ordinal logistic regression analysis after converting the antibody titer to a logged measurement (Fig. 1). The correlation coefficient between ordinal variables was calculated using Pearson's product-moment correlation coefficient. All P values were 2-sided, and P < 0.05 was considered significant.

## Results

### Patient characteristics

Patient characteristics (n = 320) are summarized in Table 1. A total of 211 patients were prescribed ICSs for bronchial asthma (n = 112, 53.1%), cough-predominant asthma (n = 24, 11.4%), and cough-variant asthma (n = 73, 34.6%). All described characteristics were significantly biased towards one group.

**Table 1:** Subjects’ characteristics

Characteristics	Inhaled corticosteroids – (n = 109)	Inhaled corticosteroids + (n = 211)	P value
Age, median, years (range)	72 (37–96)	60 (21–90)	< 0.0001
Gender Man/Woman	62/47	68/143	< 0.0001
Smoking history, yes/no	39/70	37/174	0.000474
Never smoker	70	174	
Ever smoker	28	33	
Current smoker	11	4	
Bronchial asthma, yes/no	4/105	112/99	< 0.0001
Cough predominant asthma, yes/no	0/109	24/187	< 0.0001
Cough variant asthma, yes/no	7/102	73/138	< 0.0001
Allergic rhinitis, yes/no	47/62	146/65	< 0.0001

### Antibody titer reducing factors

**Fig. 2:**
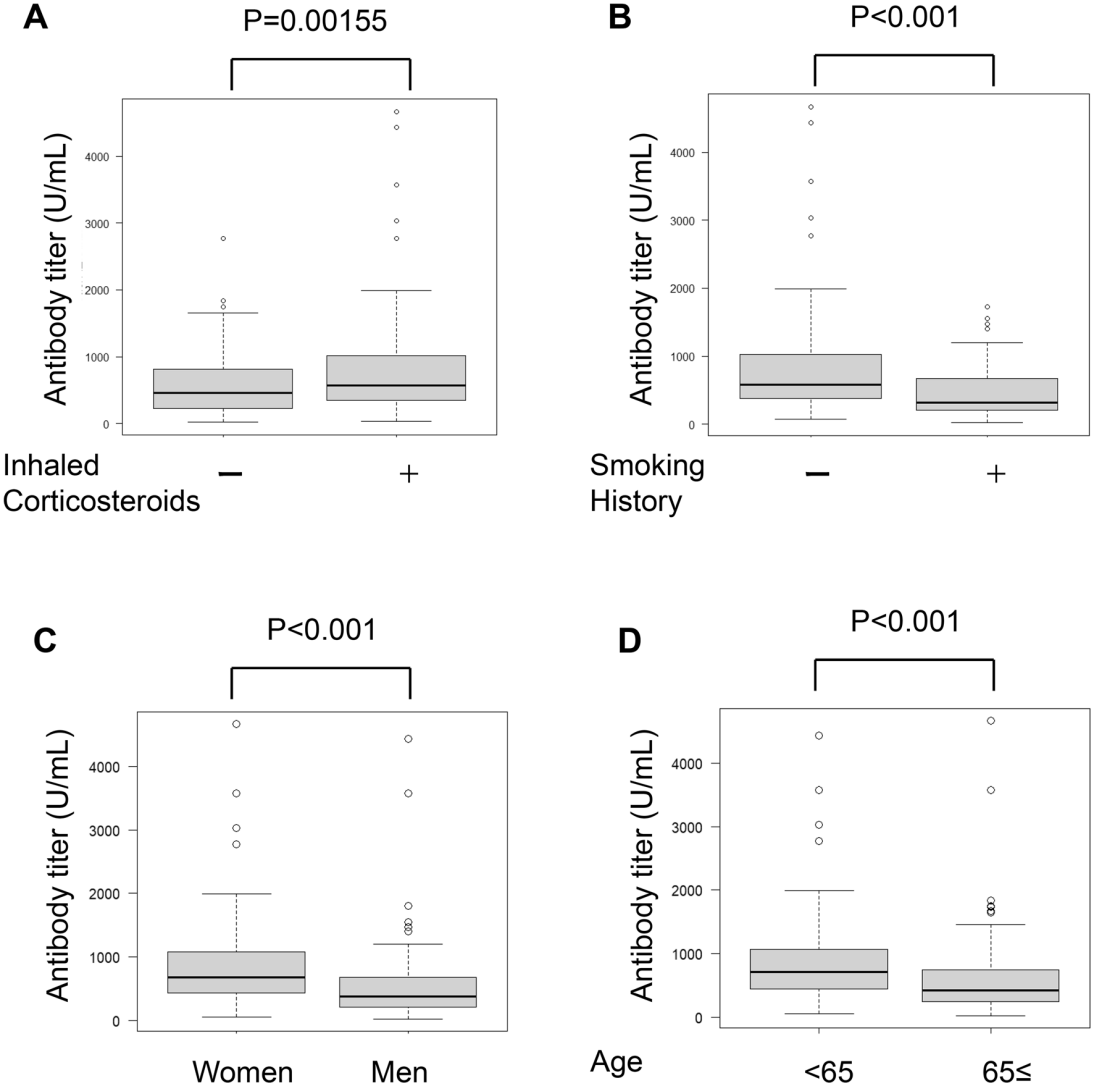
The Mann Whitney U test shows that the usage of ICSs did not reduce the antibody titer (**A**), while smoking history (**B**), elderly (**C**) and man (**D**) significantly reduced the antibody titer (all P < 0.001)

First, we investigated the relationship between ICS use and the antibody titer. The median antibody titer of those who used ICSs was 572 U/mL. This was significantly higher than the titer of 454 U/mL in patients who did not use ICSs (P = 0.00258) (Fig. 2A). Interestingly, the antibody titers of patients with smoking history were significantly lower than those of patients without a smoking history (P < 0.001) (Fig. 2B). Consistent with previous studies, the antibody titers of elderly individuals aged 65 years or older and men were significantly lower than those of individuals < 65 years of age and women (P < 0.001 and P < 0.001, respectively) (Fig. 2C, D). The median antibody titer of patients with cough-variant asthma was 706 U/mL, which was significantly higher than the tier of patients without cough-variant asthma (494.5 U/m, P = 0.000104). In contrast, there was no significant difference between antibody titers and other characteristics including bronchial asthma (P = 0.171), cough-predominant asthma (P = 0.278), and allergic rhinitis (P = 0.102).

### The impact of ICSs doses on immunogenicity

**Fig. 3:**
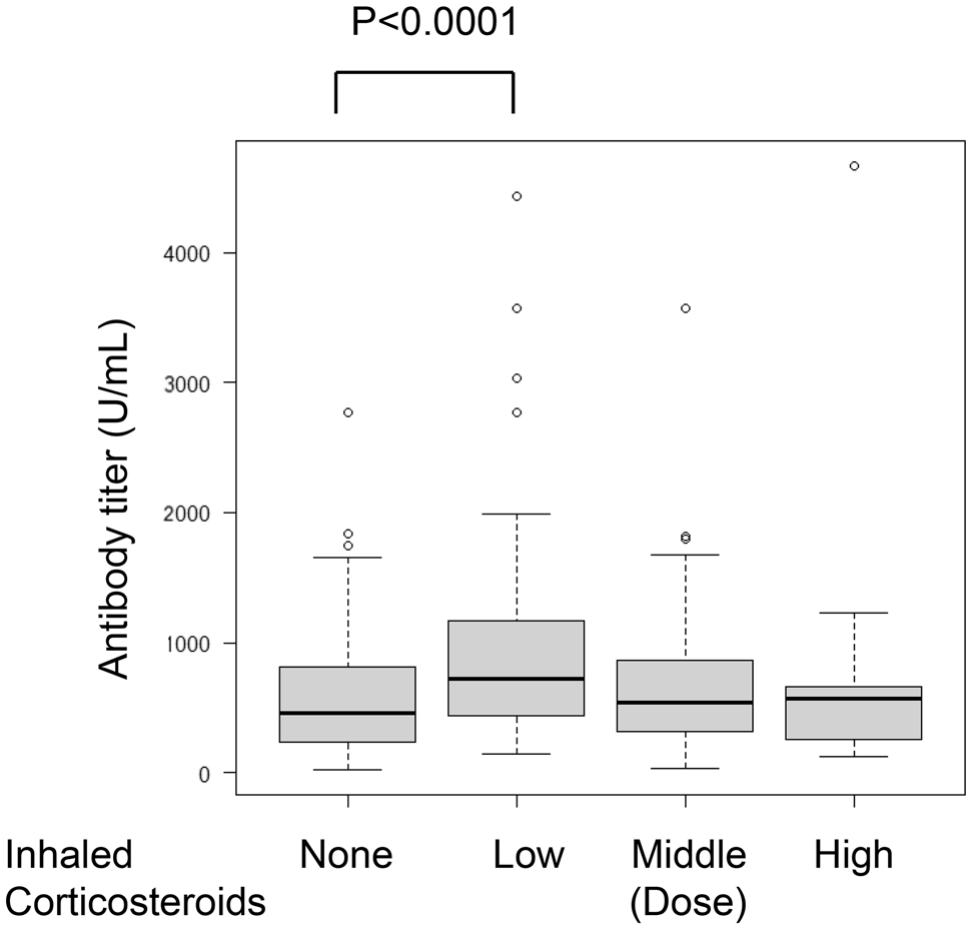
Immunogenicity is dose-dependently affected by ICS usage

Our results suggest that there was no difference in antibody titer between none, middle and high dose (Fig. 3).

### Correlation between the antibody titer and age or Brinkmann Index

**Fig. 4:**
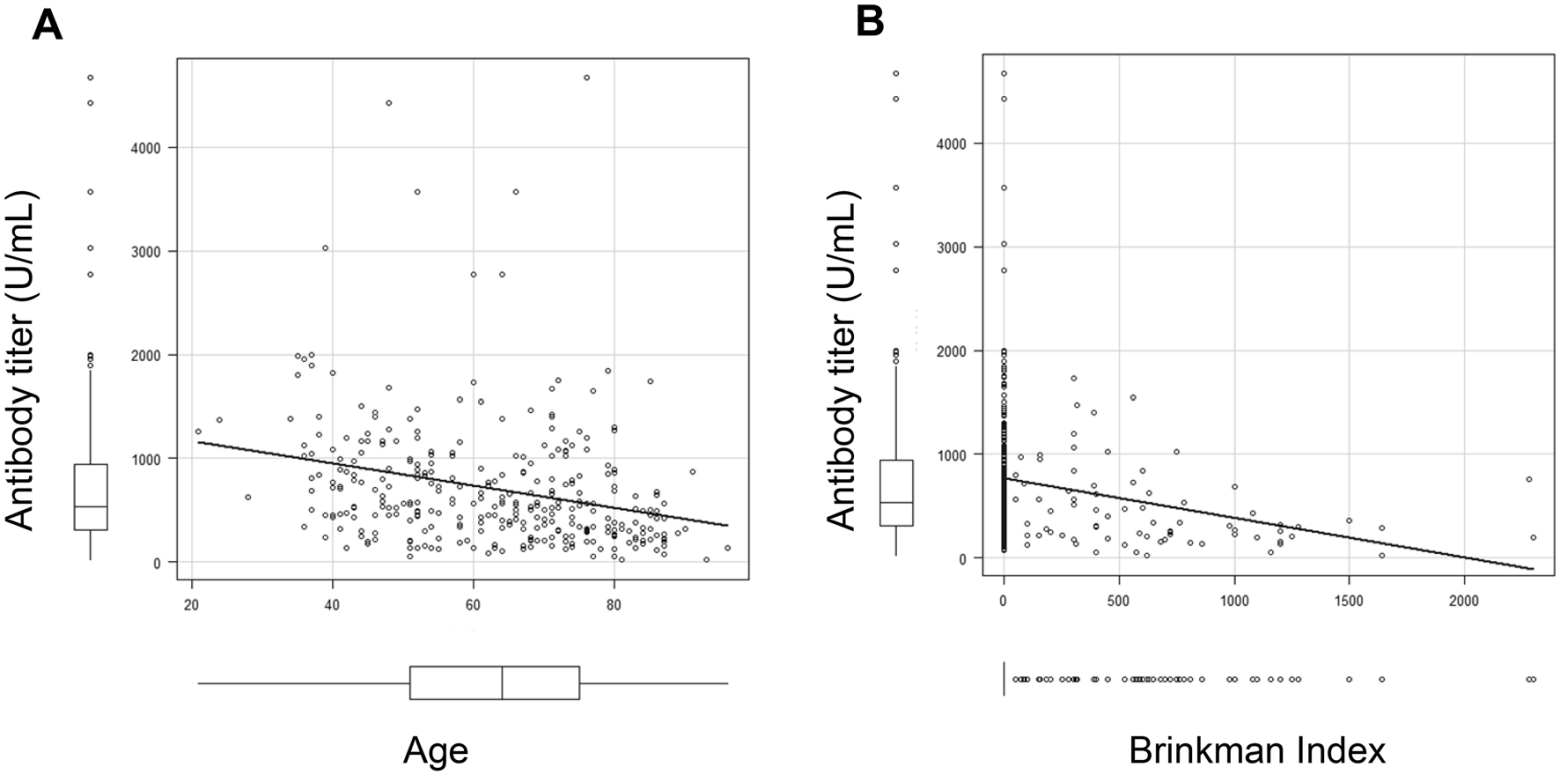
The Pearson correlation measures the strength of the linear relationship between antibody titer and age (**A**) or Brinkman Index (**B**)

Pearson’s correlation coefficient between the antibody titer and age demonstrated that the higher the age, the lower the antibody titer at-0.271 (95% confidence interval [CI] − 0.369 to − 0.166, P < 0.0001) (Fig. 4A). We also calculated the Pearson’s correlation coefficient between the antibody titer and the Brinkman index, and found that the higher the Brinkman index, the lower the antibody titer at − 0.224 (95% CI − 0.326 to − 0.117, P < 0.0001) (Fig. 4B).

### Significant antibody titer reducing factors

The multivariate analysis revealed that ICS was not an independent antibody reducing factor (P = 0.868). CVA tended to be an antibody-elevating factor (P = 0.0613) and smoking history tended to be an antibody-reducing factor (P = 0.0526). As expected, age 65 years or older (P < 0.0001) and males (P = 0.0001) were independent antibody-reducing factors (Table 2).

**Table 2:** Multivariate analysis of subjects’ features

	Odds ratio (95% confidence interval)	P value
Inhaled corticosteroids	1.040 (0.668–1.610)	0.868
Smoking history	0.598 (0.355–1.010)	0.0526
Man	0.363 (0.230–0.569)	0.0001
Aged 65 and over	0.434 (0.287–0.654)	< 0.0001
Cough variant asthma	1.570 (0.980–2.510)	0.0613

## Discussion

The antibody titers of ICSs can be affected by age, sex, and smoking. Jubishi et al. reported that self-reported asthma was correlated with elevated anti-Spike IgG levels [11]. In our study, we did not show the increase of antibody titer in asthma patients. This is partly because we excluded the patients with CVA from BA whose antibody titers were significantly higher than patients without CVA (P = 0.000104). Therefore, we performed multi-variate analysis including CVA, age, sex, and smoking and confirmed that ICSs did not have antibody-reducing effects. To the best of our knowledge, this is the first study to analyze the effects of ICSs on COVID-19 vaccination antibody titers. In the present study, we revealed that individuals aged 65 years and over and men were independent vaccine titer reducing factors. Our results are consistent with the literature [12–15]. Several studies have focused on the effects of age and sex on immune responses to vaccination [16, 17]. Giefing-Kröll et al. suggested that hormonal changes associated with aging and sex may affect the immune response to vaccination [16]. Klein et al. predicted that immunological, hormonal, genetic, and microbiota differences between men and women may also affect antibody titers [17]. Some studies have considered the possibility that the serological assay used for analysis may affect the immune response to vaccination [18]. Importantly, the quantification method for antibody titers used in this study was not identical to the ones used in previous studies.

Other than intrinsic host factors, such as age, sex, and comorbidities, extrinsic factors, including smoking history, have been analyzed in several studies [13]. Using a multivariate analysis, Costa et al. showed that significant independent vaccine titer reducing factors were elderly, males, current smoking, immunodeficiency, recent occupational contacts, and an increasing time-lapse from vaccination [18]. Ferrara et al. performed a systematic review of epidemiological studies and revealed that tobacco smoking is a significant antibody titer reducing factor in 17 out of 23 studies [19]. Some studies have shown that smoking alters immune cell counts and induces the production of inflammatory cytokines and chemokines. Smoking-induced chronic inflammation downregulates CD4^+^ T and B cells. Therefore, smoking decreases the production of IgA, IgG, and IgM [19].

In a previous study, Hanania et al. revealed that ICSs did not affect the immune response to the A antigens of the inactivated influenza vaccine in patients with asthma. However, high-dose ICSs slightly reduced the response to the B antigen of the vaccine [20]. These results concurred with our findings.

One of the study limitations is that we did not investigate the neutralizing activity. However, it was reported that there is a correlation between the neutralizing antibody titer and the antibody titer against the S protein receptor-binding domain (RBD) [21].

## Conclusions

Our results showed that ICS usage does not lower the COVID-19 vaccination antibody titer. Based on the recommendations from the GINA2021 guidelines, proper asthma management centered on ICSs is important and should be continued even during the COVID-19 pandemic.

## Data Availability

The authors will provide data upon reasonable request.

## Abbreviations

CI: Confidence interval
COVID-19: Coronavirus disease 2019
GINA: Global Initiative for Asthma
ICSs: Inhaled corticosteroids
